# Effective approaches towards teaching human anatomy among MBBS students in India

**DOI:** 10.6026/973206300221091

**Published:** 2026-02-28

**Authors:** G Krishna Kishore, Vinodhini Periyasamy, KS Deepa, J Caroline Sangeetha, B Mohamed Ismail, P Ninganagouda, C Shivaleela

**Affiliations:** 1 Department of Anatomy, Shridevi Institute of Medical Sciences and Research Hospital Tumakuru-572106, Karnataka, India; 2 Department of Anatomy, Karpagam Faculty of Medical Sciences and Research, Coimbatore, Tamilnadu, India; 3 Department of Anatomy, Anna Medical College, Mauritius, University of Technology, Mauritius; 4 Department of Anatomy, Anna Gowri Medical College and Hospital, Parameshwaramangalam, Puttur, Tirupathi, Andhra Pradesh, India; 5 Department of Community Medicine, Shridevi Institute of Medical Sciences and Research Hospital, Tumakuru-572106, Karnataka, India

**Keywords:** Early clinical exposure (ECE), first MBBS curriculum, human anatomy, medical education, self-directed learning (SDL), teaching methodologies

## Abstract

Medical anatomy forms the prime foundation in the field of clinical medicine. Observational, descriptive, qualitative analysis using questionnaire was carried out for first MBBS students at the department of Anatomy. Preference for both traditional cadaveric teaching and modern teaching techniques were appreciated among the medical students. A statistically significant proportion of students (p < 0.001) believed that, small group interactive sessions and early clinical exposure were essential for coping with hybrid PBL curriculum. Data the necessity to adopt the different teaching modalities for implementation in the modern era with the aid of available technologies.

## Background:

Medical anatomy forms the foundation pillar in the field of clinical medicine. A clear understanding of anatomy stirs a deeper interest among the young medical learners. Applying the basic knowledge of anatomy helps to improve their clinical efficiency during diagnosis and treatment strategies [1,2]. In the current era, with advanced educational technologies, effective teaching of medical anatomy for the first MBBS seems to be an uphill challenging task [3]. Competency of health professionals is the need of the hour due to global changes in the medical field. As necessity is the mother of invention, alternative medical education methodologies became inevitable over the conventional cadaveric teaching [4,5]. Conceptualisation of the human anatomy will be beneficial to advance the intricate knowledge in the basic sciences and will enhance the medical student's skill in integration with clinical sciences [6]. The revised curriculum of Medical Council of India (MCI)/ National Medical Commission (NMC) dictates revision in the traditional teaching-learning methods. NMC advised to implement a holistic approach towards interactive sessions and Self-directed learning (SDL) by introducing more e-resource based learning methods [7]. SDL in anatomy is defined as learning anatomy by oneself with suitable methods, where in, he/she takes the complete responsibility of planning and executing the learning strategies. SDL aims at moulding the student's medical profession, for an all-time learning [7,8]. Our current study aims at assessing the different effective methodologies of human anatomy for first MBBS students from our institution. Therefore, it is of interest to assess the different student's attitude, perception and feedback on the teaching and learning methods implied as per the medical curriculum.

## Materials and Methods:

## Study design and ethical approval:

An observational, descriptive, qualitative analysis was carried out at the Department of Anatomy. Ethical approval was obtained from the Institution's Ethics Committee. Informed consent was obtained from the student population.

## Sampling:

100 first-year MBBS (phase one MBBS) students (2023-2024 batches) have participated in this study. The study sample consist of 53 male and 47 female participants (n=100). Every student participated with full enthusiasm and completed the questionnaire. The participant's age ranged between 18 - 23 years. An hour was provided to complete the questionnaire study. Students also had the opportunity to express their opinion at the comments column, provided at the end of the questionnaire. The completed sheets were collected from individual student and preceded for statistical analysis.

## Statistical analysis:

Data was entered in the MS Excel sheet and statistically analysed with SPSS version 22.Statistical tests used were descriptive statistics for percentage and graphs, Chi Square test for categorical data and Bi-nominal test used for dichotomous (Yes/ No). This study will measure the student's perception on their SDL capability.

## Results and Discussion:

**Figure 1:**
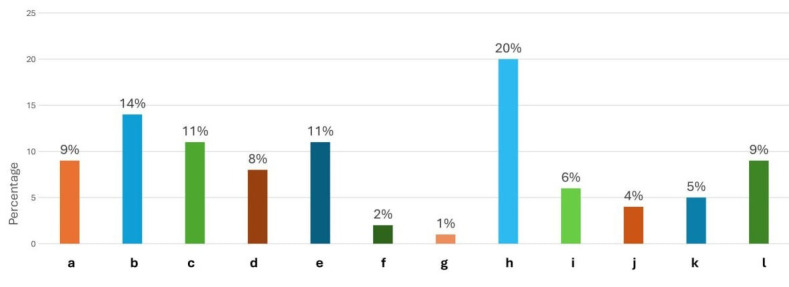
Bar diagram displays the suggestions for improving the quality of anatomy teaching - a) Increase in the hours of region-wise teaching; b) Clinical scenario based discussions after each topic; c) Use of multidimensional AV embryology teaching; d) Provide frequent quizzing and MCQs; e) Detailed diagrammatic teaching of cross sectional anatomy; f) Usage of special stained histology slides; g) Explain karyotyping in detail; h) Frequent implementation of SGL; i) Improvement in student mentoring; j) Teaching region-wise neuroanatomy; k) No comments. Satisfied with teaching methods; l) Required better vertical and horizontal integrated teaching.

First year MBBS students' opinion was taken, regarding the effective teaching-learning methods (T-L) in human anatomy by providing a framed questionnaire. The results were distributed as follows in Table 1 and Figure 1.

From the present study results, students preferred both traditional cadaveric teaching and modern teaching techniques. The current study strongly supported SDL and small group interactions of the hybrid PBL curriculum. In our present study, majority of the students favoured interactive and visually stimulating learning methods. Remarkably, 54% of students chose 3D models as their preferred classroom tool for understanding anatomy, followed by blackboard/whiteboard methods (32%). Regarding the learning patterns, kinesthetic (43%) and visual methods (33%) were favoured instead of auditory approaches. Despite the fact, digital software and mobile apps were widely preferred (42% continuously, 43% seldom), 35% of students opined to restore information in hardcopy notebooks and 35% benefit from museum resources like bones and models. A statistically significant proportion of students (p < 0.001) believed that, small group interactive sessions and ECE were essential for reiterating anatomy knowledge during clinical postings. 97% of respondents opined, ECE provided holistic knowledge to case-based questions and 93% benefitted from applying anatomical concepts to clinical scenarios. Also, students emphasized the faculty's contribution through academic guidance, mentoring and personal attention. 92% of respondents appreciated the integrating anatomical teaching coupled with basic and clinical sciences. It shows a stronger preference for both vertical and horizontal curriculum methodologies. Besides, 92% of respondents provided a positive and enthusiastic attitude towards novel learning patterns.95% strongly agreed upon interactive T-L methods. However, opinions on the clinical significance of plastination varied, new tools like 3D animation and embryoplastination were acknowledged.

The present study proves clinical integration of anatomy sessions are preferred over traditional teaching concepts. Our study findings highlighted the necessity for curriculum based integrated teaching, small group learning, ECE and 3D visualization. In a south Indian study, done by Padwal *et al.* (2025), students preferred SDL for improving anatomical skills. Students opined, lectures aided in selecting study topics and learning for the tutorial cases [6]. In an Indian study done by Souza *et al.* (2020), revealed a higher preference forcadaveric dissection hall teaching [8]. A study by Wang *et al.* (2021) also suggested cadaveric study pattern and computer assistance aids in better understanding [9]. Pradhan *et al.* (2024) opined, Indian students suggested power point presentation (PPT) over the traditional black board teaching [10]. International studies done by Abdullah *et al.* (2021), concluded best teaching methodology relied on faculty assisted human cadaveric concepts. He also summarised, prosectionsaided with clinical tutorials and electronic resources delivers a better clarity in 3Danatomy [11]. Medical youngsters learning abilities can be enhanced by engaging them in multiple modality teaching. Web based integrated approaches using imaging techniques as xeroradiography, magnetic resonance imaging (MRI) and computerised tomography scan (CT) and cone beam computed tomography (CBCT) showed a better understanding of human anatomy. Visual and auditory cognition improves the long-term memorisation and retention of anatomical concepts [10,11- 12]. Current medical education curriculum promotes problem-based learning, clinical scenario-based learning, simulated histology slides discussion, webinars, Google classroom teaching modalities with AV media to gain a solid foundation for developing professional skills [13,14]. Peer teaching distributes a wider platform for teaching and self-learning anatomy. Group discussions encourage the younger minds to explore the wide spectrum of human anatomy. Introduction of formal peer teaching in the phase-1 medical curriculum improves the visualisation of human anatomy [15]. Multiple teaching methodologies improve the communication skills in the early years of MBBS. It provides a practical understanding and memorizing gross anatomy. Plastinatedcadaveric models are preferred as secondary option due to its advantage of being formalin free, easy handling and preservation [16, 17 and 18]. E-learning is the novel practice in medical education, as electronic technologies are advocated for smarter learning. Online learning and SDL practices in developing countries like India will widen the subject excellence and favours a broad teaching opportunity for the anatomy fraternities [18, 19 and 20]. The novelty of the present study shows the different perspectives towards learning skills of the medical students in our institution. The present study results conveyed the necessity to adopt the different teaching methodologies for implementation in the modern era with the aid of available technologies.

## Conclusion:

Anatomy forms the prime cornerstone in their everyday clinical and surgical practice. As per the new medical curriculum, innovative teaching proves to be an important learning tool for MBBS students. Thus the effective teaching for phase-1 medical students can be accomplished by traditional cadaveric dissection, SDL, visual aids and integrated3D visualisation using advanced interactive techniques.

## Funding source:

This research did not receive any specific grant/ funds from funding agencies in the public, commercial or not-for-profit sectors.

## Data availability statement:

This statement does not apply for this article.

## Ethics statement:

Ethical approval was obtained from the Institution's Ethics Committee. Informed consent was taken from the student participants.

## Clinical trial registration:

This research does not involve any clinical trial.

## Permission to reproduce material from other sources:

Not Applicable.

## Author contribution:

G Krishna Kishore & Vinodhini Periyasamy contribution: Effective scientific and intellectual participation for the study, preparation and draft of the manuscript. Deepa K S Contribution: Supervised the project, preparation and draft of the manuscript. Caroline Sangeetha & Mohamed Ismail contribution: Technical procedures, data acquisition, data interpretation. Ningangowda P & Shivaleela C: Data analysis and data interpretation, critical review and final approval.

## Figures and Tables

**Table 1:** Perceptions and preferences of medical students on effective teaching-learning methods in human anatomy

**Sl. no**	**Perception of students on effective T-L methods in human anatomy**	**Objectives**	**n**	**%**	**Test Statistics(value)**	**p-value**
1	Class room methodology preferred for understanding anatomy	a) Blackboard/White board	32	32	Chi square (102.30)	0.001*
		b)Lectures	9	9		
		c)PowerPoint Presentation	5	5		
		d)3D models	54	54		
		e)OHP sheets	0	0		
2	virtual concepts favouring anatomical learning	a) Visuals (Images, videos)	33	33	Chi square (59.60)	0.001*
		b)Kinesthetics (Hands on, practicals)	43	43		
		c)Auditory (lectures, audio recordings displayed on monitor)	3	3		
		d)Interactive (quiz, games, spotters)	14	14		
		e)Group viva	7	7		
3	study patterns used for memorizing anatomy	a) Class notes	18	18	Chi square (16.90)	0.002*
		b)Flashcards / online resources	10	10		
		c)Mind maps and pneumonic	20	20		
		d)Group study	17	17		
		e) Using physical resources like bones, models etc.	35	35		
4	Storage methods for anatomy study materials	a)Physical notebooks	69	69	Chi square (114.64)	0.001*
		b)Digital folders	24	24		
		c) Flash drives	5	5		
		d)Cloud storage	2	2		
5	Usage of mobile apps / soft wares for anatomy	a) Always	42	42	Chi square	0.001*
		b)Occasionally	43	43	-49.04	
		c)Seldom	7	7		
		d)Never	8	8		
6	Hurdles encountered during preparation for anatomy exam	a)Difficulty in understanding the concepts	16	16	Chi square (51.36)	0.005*
		b)Lack of time for preparation	54	54		
		c)Less motivation	6	6		
		d)Insufficient resources	24	24		
7	Mode of support from anatomy faculties	a) Study groups	17	17	Chi square (6.00)	0.112#
		b)Personal mentoring	26	26		
		c)Academic advices	23	23		
		d)More time to be spent individually	34	34		
8	Scores for effectiveness in current anatomy education	a)1= Poor	5	5	Chi square (34.96)	0.001*
		b)2= Moderate	36	36		
		c)3= Consistent	42	42		
		d)4= Very good	17	17		
9	Rate the necessity of technology aided learning tools for learning anatomy	a)1= Not effective	18	18	Chi square (4.40)	0.006*
		b)2= Moderately effective	31	31		
		c)3= Consistent	22	22		
		d)4= Very effective	29	29		
10	Feasibility in applying anatomy concepts with the clinical scenarios	a) Yes	93	93	Binomial (73.96)	0.002*
		b)No	7	7		
11	preferable methodology in engaging practical sessions	a)Small group oriented interactive sessions	99	99	Binomial (96.04)	0.001*
		b) Classroom recorded lectures	1	1		
12	Do you find early clinical exposure (ECE) to be supportive in better understanding anatomy?	a)Yes	99	99	Binomial (96.04)	0.001*
		b)No	1	1		
13	With the help of ECE sessions,	a) Yes	97	97	Binomial (88.36)	0.005*
	Can you answer the clinical based scenario questions?	b) No	3	3		
14	Are you able to appreciate the integrated teaching of anatomy with other basic sciences?	a)Yes	92	92	Binomial (70.56)	0.001*
		b)No	8	8		
15	Are you able to escalate the integrated teaching of anatomy with other clinical sciences?	a)Yes	92	92	Binomial (70.56)	0.001*
		b)No	8	8		
16	Implementation of Seminars / SDL / Conceptual learning helps in	a) Understanding the topic	20	20	Chi square (7.92)	0.048*
		b)Gaining knowledge and interest	35	35		
		c)Develops communication skills	17	17		
		d)Integrate the knowledge with the clinical concepts	28	28		
17	Did you gain ample knowledge, skills and attitude towards the medical setup from your anatomy learning?	a)Yes	36	36	Chi square (40.88)	0.002*
		b)No	6	6		
		c)Partially	58	58		
18	Does a deeper understanding of anatomy improve your problem-solving skills in clinical scenario?	a)Completely agree	50	50	Chi square (63.76)	0.001*
		b)Moderately	39	39		
		c)Partially agree	8	8		
		d)Poor agreement	3	3		
19	Qualities expected from an anatomy faculty	a) Impart micro skills framework	6	6	Chi square (61.92)	0.005*
		b)Develops a good teacher-student rapport	18	18		
		c)Provides a lifelong learning skill	18	18		
		d)Simplify tougher topics and provide clear concepts	58	58		
20	Do interactive teaching-learning methods are significant in developing critical thinking?	a)I strongly agree	95	95	Binomial (81.00)	0.001*
		b)I disagree	5	5		
21	Do you agree, it is pertinent to have an enthusiastic and knowledgeable attitude towards learning anatomy?	a) 1= Not important	4	4	Chi square (91.28)	0.001*
		b)2= Partially important	10	10		
		c)3= Moderately important	21	21		
		d)4= Strongly important	65	65		
22	Do you support plastination models in anatomy to correlate with real life scenario in clinical setup?	a) Yes	38	38	Binomial (5.76)	0.021*
		b)No	62	62		
23	Understanding embryology will be effective with	a)Blackboard teaching	12	12	Chi square (81.20)	0.001*
		b)Plastination models	13	13		
		c)Clay models	11	11		
		d)3 D animation display	64	64		
24	Do you appreciate discussing the clinical case scenario before teaching the particular anatomy topic	a)Yes	93	93	Binomial (73.96)	0.005*
		b) No	7	7		
25	Suggestions for improvement in the quality of teaching	a)Detailed teaching and increase the hours of teaching-region wise	9	9	Chi square (37.52)	0.033*
		b)Clinical scenario based discussion at the end of each topic	14	14		
		c)Use of multidimensional AV embryology teaching	11	11		
		d)Provide frequent quizzes and MCQs for a broad understanding	8	8		
		e)Detailed diagram teaching cross sectional anatomy	11	11		
		f)Usage of special stained slides	2	2		
		g)Explain karyotyping in detail	1	1		
		h)Frequent implementation of SGL	20	20		
		i)Improvement in mentoring the subject with attention towards individual student	6	6		
		j)Teaching neuroanatomy with region wise models will be productive and easy to remember	4	4		
		k)No comments, satisfied with the curriculum and teaching methods	5	5		
		l)Required more vertical and horizontal integrated teaching sessions	9	9		
Statistical analysis test used;
Chi square test and Binominal test.
* showing p value <0.05 indicates statistically significant,
# showing p value >0.05 indicates statistically not significant
